# Increased Levels of Circulating IL-16 and Apoptosis Markers Are Related to the Activity of Whipple's Disease

**DOI:** 10.1371/journal.pone.0000494

**Published:** 2007-06-06

**Authors:** Marie Benoit, Florence Fenollar, Didier Raoult, Jean-Louis Mege

**Affiliations:** Unité des Rickettsies, Centre National de la Recherche Scientifique Unité Mixte de Recherche 6020, Institut Fédératif de Recherche 48, Université de la Méditerranée, Faculté de Médecine, Marseille, France; Federal University of Sao Paulo, Brazil

## Abstract

**Background:**

Whipple's disease (WD) is an infectious disease caused by *Tropheryma whipplei*, which replicates in macrophages and induces the release of interleukin (IL)-16, a substrate of caspase 3, and macrophage apoptosis. The disease is characterized by intestinal, cardiac or neurological manifestations; its diagnosis is based on invasive analysis requiring tissue biopsies or cerebrospinal fluid puncture. The disease progression is slow and often complicated by relapses despite empirical antibiotic treatment.

**Methodology/Principal findings:**

We monitored circulating levels of IL-16 and nucleosomes in 36 French patients with WD; among them, some patients were enrolled in a longitudinal follow-up. As compared to control subjects, the circulating levels of both IL-16 and nucleosomes were increased in untreated patients with WD presenting as intestinal, cardiac or neurological manifestations. This finding was specific to WD since the circulating levels of IL-16 and nucleosomes were not increased in patients with unrelated inflammatory diseases such as inflammatory bowel disease or Q fever endocarditis. We also found that increased levels of IL-16 and nucleosomes were related to the activity of the disease. Indeed, successful antibiotic treatment decreased those levels down to those of control subjects. In contrast, patients who suffered from relapses exhibited circulating levels of IL-16 and nucleosomes as high as those of untreated patients.

**Conclusions/Significance:**

Circulating levels of both IL-16 and nucleosomes were increased in WD. This finding provides simple and non-invasive tools for the diagnosis and the prognosis of WD.

## Introduction

Whipple's disease (WD) is a systemic disease, first described in 1907 by the American pathologist George H. Whipple as an intestinal lipodystrophy [1]. WD, with fewer than 1,000 cases reported to date, is considered to be rare although its incidence is probably underestimated [2]. WD has been traditionally seen as a gastrointestinal disease characterized by polyarthritis, fatigue, weight loss, and anemia, followed by a progressive syndrome of abdominal pain, distension, steatorrhea, and severe cachexia [3]. In about 15% of reported cases, gastrointestinal symptoms are lacking, and the disease appears as cardiac manifestations such as myocarditis, pericarditis and negative blood culture endocarditis [4], or as neurological manifestations including dementia, lethargy and neurological deficits [5]. The evolution of WD is chronic with relapses despite empirical antibiotic treatment. The bacterial etiology of WD was first established in 1961 by the detection of “bacillary bodies” in the intestine of patients [6]. The causative agent of WD was identified in 1992 as a gram-positive bacterium, phylogenetically close to the Actinobacter clade as determined by a molecular approach [7]. In 2000, it was isolated from a patient with WD and successfully cultured [8]. In 2001, the name of *Tropheryma whipplei* was officially ascribed to the WD agent [9], and the complete sequencing of two strains of *T. whipplei* was performed in 2003 [10], [11].

The diagnosis of WD has been based for many years on the presence in intestinal biopsies of large, foamy macrophages containing periodic acid-Schiff (PAS)-positive inclusions in the lamina propria, but these PAS-positive inclusions may also be detected in other tissues [3]. Although the recent development of molecular tools has improved bacterial detection in tissues [12], the diagnosis of WD remains invasive [13]. The current treatment is trimethoprim-sulfamethoxazole given for at least one year [14]. The antibiotic treatment of WD is empirical and the choice of drug and the duration of treatment are controversial. The risk of clinical relapses is high; patients typically have primary or recurrent clinical manifestations after arrest of treatment, especially those patients presenting with neurological manifestations [3]. Finally, invasive investigations are required at least every six months after diagnosis to assess the response of patients with WD to antibiotic treatment [3].

In WD, macrophages present in intestinal lesions exhibit an anti-inflammatory transcriptional profile and a pro-apoptotic program [15]. In human monocyte-derived macrophages, *T. whipplei* stimulates the release of interleukin (IL)-16 that is critical for bacterial replication, and induces macrophage apoptosis [16]. IL-16 is synthesized as a precursor of 69 kDa, named pro-IL-16, which is a substrate for caspase 3, the central effector of apoptosis. The cleavage of pro-IL-16 by caspase 3 releases the biologically active form of the molecule, which consists of a secreted fragment of 56 kDa [17]. IL-16 is an immunomodulatory cytokine released at the inflammatory site. Through its interaction with CD4, IL-16 acts as a chemoattractant for CD4^+^ immune cells including T-cells, monocytes and eosinophils [18]. IL-16-expressing cells include mononuclear phagocytes [19], CD4^+^ [20] and CD8^+^ T-cells [21], eosinophils [22] and mast cells [23]. In preliminary experiments, we have shown that circulating levels of IL-16 are increased in some patients with WD [16]. In this study, we examined whether IL-16 and apoptosis markers were increased in patients with WD. Increased circulating levels of IL-16 and nucleosomes were present in patients with WD before the beginning of their treatment. Antibiotic treatment decreased the levels of both circulating IL-16 and nucleosomes, whereas patients who relapsed exhibited similar levels of IL-16 and nucleosomes to those of untreated patients. We suggest that IL-16 and nucleosomes may be useful to assess the prognosis for and the response to treatment in patients with WD.

## Methods

### Patients

Thirty-six French patients with WD (27 men and 9 women) were included in the study after giving informed consent and receiving approbation by the Ethics Committee of the Université de la Méditerranée. The diagnosis was based on clinical features, histological findings, PCR studies and cultures of tissue samples [24]. The criteria for confirming the diagnosis of classic Whipple's disease and endocarditis due to *T. whipplei* were previously described [25], [26], [27]. The criteria for confirming the diagnosis of isolated neurological manifestations due to *T. whipplei* included two positive PCR assays targeting two different genes performed on two different cerebrospinal fluid samples. The criteria for confirming the diagnosis of uveitis due to *T. whipplei* included one positive PCR assay targeting two different genes performed on aqueous humor specimen. For each PCR, positive and negative controls were used. The features of these patients are detailed in Table 1. The control groups consisted of healthy subjects (5 women and 8 men), and patients with unrelated diseases. Six patients with inflammatory bowel disease (3 men with Crohn's disease and 3 women with ulcerative colitis) were included. The diagnosis of inflammatory bowel disease was established by a combination of clinical evaluation with endoscopic, histological, radiological, and/or biochemical investigations after exclusion of enteric infections and ischemia [28]. As controls against infectious endocarditis, we included in the study 7 patients (3 men and 4 women) with Q fever endocarditis, an endocarditis caused by *Coxiella burnetii*, which is an intracellular bacterium that specifically infects macrophages [29]. The diagnosis of Q fever endocarditis was based on the modified Duke's University criteria including the presence of phase I *C. burnetii*-specific IgG [30]. Finally, we selected 13 men without WD but with stool or saliva PCR positive for *T. whipplei* [31], [32], who were presented in the study as asymptomatic.

**Table I:** Epidemiological and clinical features of patients with WD

Presentations of Whipple's disease	Males/females	Age Mean [Range]	Diagnosis	Main clinical manifestations
Classical Whipple's disease	15/9	62 [35–78]	PAS^+^, IHC^+^ and PCR^+^ on duodenal biopsies	Diarrhea, arthralgia, fever, WL, AP, adenopathy, pleuritis, asthenia, myalgia, ascitis, pigmentation
		48 [33–73]		
Endocarditis due to *T. whipplei*	5/0	60 [51–67]	PAS^+^, IHC^+^ and PCR^+^ on cardiac valves	Arthralgia, WL, dyspnea, arterial embolus, and/or stroke
			PAS^−^, IHC^−^ and PCR^−^ on duodenal biopsies	
Isolated neurological manifestations due to *T. whipplei*	6/0	47 [36–59]	PCR^+^ on cerebrospinal fluid for all patients	WL, asthenia, cognitive troubles, myoclonus, personality changes, and/or hypothalamic manifestations
			PCR^+^ and PAS^+^ on brain biopsy for one patient	
			PCR^+^ on stools for two patients	
			PCR^+^ on saliva for one patient	
			PAS^−^, IHC^−^ and PCR^−^ on duodenal biopsies	
Uveitis due to *T. whipplei*	1/0	80	PCR^+^ on aqueous humor	Uveitis
			PCR^+^ on stools	
			PAS^−^, IHC^−^ and PCR^−^ on duodenal biopsies	

Footnotes: PAS = Periodic acid-Schiff staining, IHC = Immunohistochemistry performed using polyclonal rabbit anti-*T. whipplei* antibodies, PCR = Polymerase chain reaction, WL = Weight Loss, AP Abdominal Pain

### Determination of circulating IL-16

Blood was collected on EDTA tubes and centrifuged 15 min at 300 *g*. Plasma was collected and stored at −80°C. Plasma levels of bioactive IL-16 were measured by ELISA assay (R&D Systems), according to the manufacturer's recommendations. The detection limit was 6.2 pg/ml. The intra- and inter-specific coefficients of variation ranged from 5 to 10%.

### Determination of circulating apoptosis markers

Plasma levels of nucleosomes were measured using an ELISA cell death detection plus kit (Roche Diagnostics). This assay is based on a quantitative sandwich enzyme immunoassay that recognizes DNA and histones [33]. The specific enrichment factor in nucleosomes, expressed in arbitrary units, was calculated according to the manufacturer's instructions.

Caspase activity was measured with the Apoptosis Detection Polycaspase Assay Kit (Immunochemistry Technologies), according to the manufacturer's protocol. The assay is based on a Fluorochrome Inhibitor of Caspases (FLICA) that binds covalently active caspases in the cells. Briefly, 100 µl of whole blood were incubated in 96-wells with 5 µL of 30X FLICA solution for 1 h at room temperature and washed twice. Red blood cells were then lyzed with 150 mM ammonium chloride for 10 min under agitation. Leukocytes were centrifuged 10 min at 400 *g*, washed twice, fixed and analyzed by flow cytometry (EPICS XL, Coulter). Gating was performed using forward and side scatter to remove dead cells and remaining red blood cells from the analysis. Fifty thousand events were acquired for each sample. The percentage of leukocytes with active caspases was determined using the Expo32 ADC software.

### Statistical analysis

Results were expressed as individual values with medians. Quantitative data were compared with the non-parametric Mann-Whitney *U* test. Differences were considered significant at p<0.05. The levels of sensitivity (Se) and specificity (Sp) were calculated manually: Se = (TP/(TP+FN)) and Sp = (TN/(TN+FP)), where TP: True Positives, FN: False Negatives, TN: True Negatives, and FP: False Positives.

## Results

### Circulating IL-16 and apoptosis markers in untreated patients with WD

**Figure 1:**
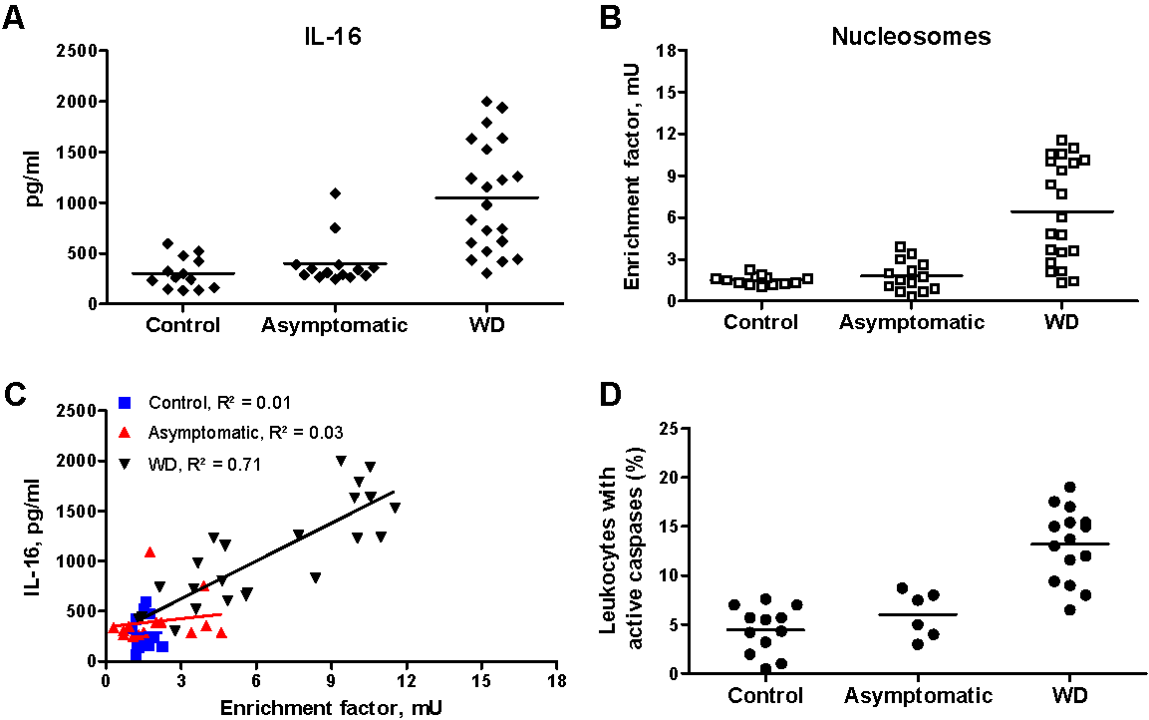
Circulating IL-16 and apoptosis markers in untreated WD patients. A and B, The circulating levels of IL-16 (A) and nucleosomes (B) were determined by immunoassays in control subjects, asymptomatic subjects and patients with WD. Results are expressed as individual values with medians. C, Correlation between circulating levels of IL-16 and nucleosomes in control subjects, asymptomatic subjects and patients with WD. Dots were analyzed by linear regression. D, The caspase activity in leukocytes (FLICA measurement) was analyzed by flow cytometry. Results are expressed as the percentage of leukocytes that expressed active caspases.

Since the bacteriological diagnosis of WD in France is established in our laboratory, the frozen plasma of individuals suspected of WD was collected over a 3-year period. Only plasmas from patients with confirmed WD were subsequently analyzed for the presence of IL-16 and nucleosomes. Some patients were seen in Marseille by our medical staff, which allowed for leukocyte study and the longitudinal follow-up of these patients. In untreated patients with WD, circulating levels of IL-16 were significantly (p<0.001) increased as compared to asymptomatic subjects and control subjects (Fig 1A). The apoptosis was investigated by measuring circulating nucleosomes and caspase activity in leukocytes. Circulating nucleosomes were significantly (p<0.001) increased in untreated patients with WD as compared to asymptomatic subjects and control subjects (Fig 1B). Circulating IL-16 and nucleosomes were related in untreated patients with WD (y = 125.73x+246.71, correlation coefficient R2 = 0.71) but were not related in asymptomatic subjects (y = 27.35x+344.60, R2 = 0.03) or control subjects (y = 0.75x+280.02, R2 = 0.01) (Fig 1C). In addition, we collected leukocytes of 15 untreated patients with WD. Active caspases in leukocytes were significantly higher (p<0.001) in untreated patients with WD (13.2±1.0%) than in asymptomatic subjects (6.1±1.0%) or control subjects (4.5±0.7%) (Fig 1D).

**Figure 2:**
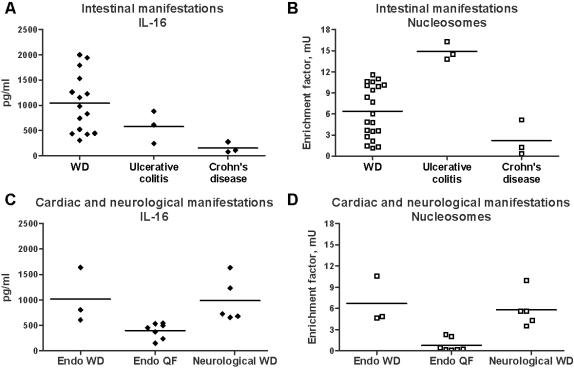
Clinical manifestations and circulating IL-16 and apoptosis markers. A and B, Circulating levels of IL-16 (A) and nucleosomes (B) in untreated patients with intestinal manifestations of WD, ulcerative colitis and Crohn's disease. C and D, Circulating levels of IL-16 (C) and nucleosomes (D) in untreated patients with cardiac (Endo WD) or neurological manifestations of WD, and Q fever endocarditis (Endo QF). Results are expressed as individual values with medians.

Although untreated patients with WD exhibited high circulating levels of IL-16 and nucleosomes, there was some heterogeneity in patients. As WD is characterized by different intestinal, cardiac or neurological manifestations, we asked if the levels of IL-16 and nucleosomes were related to the clinical manifestations of WD. Circulating levels of IL-16 and nucleosomes were similarly increased in patients with intestinal (Fig 2A and B), cardiac and neurological manifestations (Fig 2C and D). Increased levels of both IL-16 and nucleosomes were specific to WD. Indeed, untreated patients with intestinal manifestations of WD exhibited significantly higher circulating levels of IL-16 than patients with ulcerative colitis (p<0.05) or Crohn's disease (p<0.01) (Fig 2A). Circulating nucleosomes were significantly (p<0.05) higher in untreated patients with intestinal manifestations of WD as compared to patients with Crohn's disease (Fig 2B). In contrast, patients with ulcerative colitis exhibited significantly higher levels of circulating nucleosomes than untreated patients with intestinal manifestations of WD (p<0.01) or Crohn's disease (p<0.05) (Fig. 2B). As compared to the infective endocarditis due to *C. burnetii*, untreated patients with endocarditis due to *T. whipplei* exhibited significantly (p<0.01) higher levels of circulating IL-16 (Fig. 2C) and nucleosomes (Fig 2D). Taken together, these results show that high circulating levels of both IL-16 and nucleosomes characterized untreated patients with WD.

As no simple serological test currently allows the diagnosis of WD, we tested the value of IL-16 and nucleosomes for diagnosis of WD in this small sampling. Using a cut-off of 500 pg/ml for IL-16, the sensitivity of the test to detect active WD was 80% (16/20 samples) and the specificity was 85% (23/27 samples). Using a cut-off of 1,000 pg/ml, these values were 45% (10/22 samples) and 96% (26/27 samples), respectively. For nucleosomes, a cut-off of 3 mU led to a sensitivity of 76% (16/21 samples) and a specificity of 89% (24/27 samples). A cut-off of 6 mU led to a sensitivity of 52% (11/21 samples) and a specificity of 100% (27/27 samples). Using the combination of the two parameters, a cut-off of 500 pg/ml for IL-16 and of 3 mU for nucleosomes led to a sensitivity of 80% (16/20 samples) and a specificity of 96% (26/27 samples). Using a cut-off of 1,000 pg/ml for IL-16 and of 6 mU for nucleosomes, the sensitivity was 50% (9/18 samples) and the specificity was 100% (27/27 samples).

### Circulating IL-16 and nucleosomes in treated WD patients

**Figure 3:**
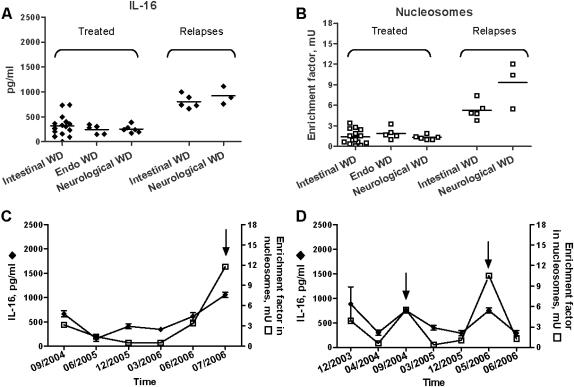
Effect of antibiotic treatment on circulating levels of IL-16 and nucleosomes. A and B, Circulating levels of IL-16 (A) and nucleosomes (B) in patients successfully treated and patients who suffered from relapses. C and D, Time course of circulating IL-16 (♦) and nucleosomes (□) in two patients with neurological manifestations of WD who suffered from relapses. ↑ represents the date of clinical relapses.

To determine if high levels of IL-16 and nucleosomes are related to active WD, we monitored circulating IL-16 and nucleosomes in 26 patients treated with antibiotics and in 8 patients with relapses despite antibiotic treatment. Antibiotic treatment decreased the circulating levels of IL-16 (Fig. 3A) and nucleosomes (Fig. 3B) in patients down to levels comparable to those found in control subjects, independently of the clinical manifestations of WD. In relapsing patients with intestinal or neurological manifestations of WD, the circulating levels of IL-16 (Fig 3A) and nucleosomes (Fig 3B) were high and similar to those found in untreated patients with WD. Finally, we regularly monitored 2 patients with neurological manifestations of WD who suffered from relapses after arrest of their antibiotic treatment. The first patient exhibited low levels of IL-16 and nucleosomes during the total duration of his treatment; few months after the arrest of his treatment, he presented clinical symptoms of relapses, and both circulating IL-16 and nucleosomes were increased (Fig 3C). The second patient presented 2 relapses. The levels of circulating IL-16 and nucleosomes were transiently increased during the clinical relapses, and decreased when clinical symptoms disappeared after newly designed antibiotic therapy (Fig 3D). These results demonstrate that antibiotic treatment of patients with WD decreased circulating levels of IL-16 and nucleosomes whereas relapses were associated with high levels of IL-16 and nucleosomes, suggesting that circulating IL-16 and nucleosomes may be used as markers of active WD.

## Discussion

We show here that systemic IL-16 is related to WD. While increased IL-16 has been reported in local lesions of chronic immune diseases, including allergen-induced bronchial asthma and rheumatoid arthritis [34] and in the mucosa of patients with inflammatory bowel disease, including ulcerative colitis and Crohn's disease [35], [36], the systemic role of IL-16 is a new finding. Indeed, circulating levels of IL-16 were not increased in patients with ulcerative colitis or Crohn's disease even though IL-16 is expressed in tissue lesions. Conversely, circulating IL-16 was increased in patients with WD (our results) and is produced by macrophages isolated from blood [16], but the expression of IL-16 is not increased in macrophages infiltrating intestinal lesions of one patient with WD [15], suggesting that IL-16 is associated with the systemic phase of WD. The presence of high circulating levels of IL-16 in WD could not be explained by the production of IL-16 by human macrophages stimulated by *T. whipplei* but evoked other cell types as a source for IL-16. Indeed, some patients with WD exhibited a high number of eosinophils [2], known to produce IL-16 [22], but the patients with hypereosinophilia did not exhibit higher levels of IL-16 (data not shown).

We also showed that systemic IL-16 was associated with apoptosis in WD. We previously reported that *T. whipplei* persistence is associated with the up-regulated expression of pro-apoptotic genes in vitro [16] and in vivo [15]. Since IL-16 was more prevalent in blood from patients with WD, we studied those patients' circulating levels of nucleosomes, which result from DNA fragmentation in the late stages of apoptosis. Circulating levels of nucleosomes were increased in untreated patients with WD and correlated with increased caspase activity in leukocytes. In addition, circulating levels of nucleosomes were correlated to those of IL-16, suggesting a clear-cut relationship between these markers. This finding may be related to the fact that IL-16 is a substrate of caspase 3, an effector of the apoptotic pathway.

Increased circulating levels of IL-16 and nucleosomes were specific to WD. Indeed, patients with ulcerative colitis exhibited very high levels of circulating nucleosomes as compared to patients with WD, but their circulating levels of IL-16 were similar to those of healthy controls. In contrast, patients with Crohn's disease or with Q fever endocarditis had low levels of IL-16 and nucleosomes. The combination of increased levels of IL-16 and caspase 3 activity has been reported in cerebrospinal fluid from patients with multiple sclerosis and experimental autoimmune encephalomyelitis, and is correlated with disease activity [37], [38]. Increased serum levels of IL-16 have been associated with atopic dermatitis in children, but are not correlated with severity of the disease [39]. In addition, increased circulating levels of IL-16 and nucleosomes were specific to WD disease, not to the *T. whipplei* infection. Indeed, subjects which were PCR positive for *T. whipplei* in their stool and saliva but who lacked clinical manifestations did not exhibit increased levels of IL-16 and nucleosomes. In a random sample of 40 healthy individuals, 35% showed evidence of *T. whipplei* DNA in their saliva [32]. *T. whipplei* DNA was found in 13% of duodenal biopsies or the gastric juice of 105 patients having elective gastroscopy, with no clinical signs of WD [31]. The detection of *T. whipplei* DNA on repeated samples suggests that *T. whipplei* may be an oral commensal organism that is ubiquitous and generally not pathogenic [40]. Using a rather small number of patients with active WD, we found that the sensitivity and the specificity of measurements of circulating IL-16 and nucleosomes have a good value for the diagnosis of active WD. However, the tested samples are small and these results, although encouraging, should be considered preliminary. It is likely that increased levels of circulating IL-16 and nucleosomes in patients with WD are a hallmark of the disease and can permit doctors to discriminate between subjects with WD and healthy carriers.

Finally, high levels of circulating IL-16 and nucleosomes were related to the activity of WD because the successful antibiotic treatment decreased both markers in patients with WD. Antibiotic therapy leads to a rapid improvement in the clinical status of the majority of patients with WD [41]. Diarrhea and fever can resolve within 1 week of the start of therapy, arthropathy and other symptoms improve after a few weeks, and a normalization of laboratory findings is observed within a few months in most patients [3]. However, these patients are followed by analysis of cerebrospinal fluid or duodenal biopsies 6 months and 12 months after diagnosis. Antibiotic treatment is generally stopped when PCR for *T. whipplei* and PAS staining are negative [3]. In some patients, the persistent bacterial infection of tissues results in relapses. The current follow-up for these patients consists of analysis of the cerebrospinal fluid or intestinal biopsies every 6 months until bacterial material is undetectable, which can require several years [2]. Patients with relapses exhibited levels of circulating IL-16 and nucleosomes as high as those of untreated patients. We suggest that the dosage of circulating levels of IL-16 and nucleosomes together with simple, rapid and non-invasive tests may be useful to check the efficiency of the antibiotic treatment and the occurrence of relapses in patients with WD.

In conclusion, persistent levels of circulating IL-16 and apoptosis markers were specifically associated with active WD, whatever the clinical manifestations of the disease. These levels and markers may provide simple and non-invasive tools for the diagnosis of and the prognosis for WD. Our results also suggest that anti-IL-16 therapy could be administered along with antibiotic treatment for patients with WD, as successfully reported in experimental autoimmune encephalomyelitis [42].
